# Allopatry, competitor recognition and heterospecific aggression in crater lake cichlids

**DOI:** 10.1186/s12862-015-0569-9

**Published:** 2016-01-04

**Authors:** Topi K. Lehtonen, Karine Gagnon, Will Sowersby, Bob B. M. Wong

**Affiliations:** Department of Biology, University of Turku, Turku, Finland; School of Biological Sciences, Monash University, Melbourne, Australia

**Keywords:** Allopatry, Behavioural plasticity, Cichlid fish, Colour signal, Competitor recognition, Heterospecific aggression, Phenotypic similarity, Signal reliability, Species interaction

## Abstract

**Background:**

Aggressive behaviour can have significant evolutionary consequences–not only within species, but also in the context of heterospecific interactions. Here, we carried out an experimental field study to investigate the importance of phenotypic similarity on levels of aggression between species whilst controlling for familiarity effects using manipulated allopatric stimuli. Specifically, we investigated aggressive responses of territory holding males and females in two species of Neotropical cichlid fish, *Amphilophus sagittae* and *Hypsophrys nicaraguensis*, that differ in their phenotypic similarity to our allopatric stimulus species, *Amphilophus astorquii*.

**Results:**

We found that, independent of phenotypic similarity (and correlated phylogenetic proximity) between the territory holders and intruder, territorial aggression was not adjusted in relation to allopatric intruder colour markings that are associated with different levels of threat and known to provoke different responses in a sympatric setting. We also found that males and females did not differ in their overall patterns of aggression adjustment towards intruder cues. Nevertheless, the two focal species, which share the same breeding grounds and external threats, exhibited different sex roles in breeding territory defence.

**Conclusion:**

Together with earlier studies assessing hetrospecific aggression in sympatry, our current results highlight the importance of coevolution and learning in species interactions.

## Background

Heterospecific aggression tends to be more pronounced among congeneric, phenotypically similar species as compared to aggression directed to those from other genera and different phenotypes [1, 2]. Besides the extent of niche overlap, competitor recognition is assumed to play a significant role in the evolution of heterospecific aggression [1, 2]. Indeed, to properly adjust its aggressive responses, a territory holder needs to be able to correctly recognize (heterospecific) intruders that pose different levels of threat [3, 4]. Here, individuals may rely on similar sensory and cognitive means for recognising phenotypically similar heterospecifics as they would for conspecifics [5]. As a consequence, it may be easier for territory holders to appropriately adjust aggression towards those heterospecifics to which they have a higher phenotypic resemblance. Likewise, a novel heterospecific signal may be easier to detect when it is similar to a familiar signal [6].

Signal recognition can be driven not only by niche overlap or phenotypic resemblance between interacting species [2, 6, 7], but also by learning opportunities. For example, blue-coloured males in *Pundamilia* cichlid fish adjust their aggression depending on their prior exposure to red (as opposed to only blue) males [8]. Such effects can also be sex-specific, as shown in female damselflies, with species recognition in a mating context being affected by their prior experience with conspecific and heterospecific males [9]. The result of a recent meta-analysis also suggests that species recognition, at least in a mating context, may have evolved quite differently between the sexes, with the capacity to discriminate between conspecifics and heterospecifcs being based more on learning in females [5]. Consistent with such an interpretation, the sexes are often subject to divergent selection pressures in terms of, for example, aggressive behaviour and parental roles [10, 11] while, at the proximate level, they may also differ in cognitive abilities in colour or pattern recognition [12, 13]. Based on such findings, it is conceivable that the sexes could also differ both in their opportunity and ability to recognise territorial intruders, a prediction that has hitherto been subject to very little empirical attention [5].

In fish, colour cues have often evolved to play an important role in species recognition, in both a competition and reproduction context. This is especially true in cichlids [14, 15]. For instance, in the Central American Midas cichlid species complex (within the genus *Amphilophus*, see [16, 17]), experiments with manipulated (i.e. ‘dummy’) stimuli have found that coloration alone is a sufficient cue for competitor recognition both within [18] and among sympatric species [19]. Earlier results on African cichlids also indicate that phenotypic similarity may affect heterospecific aggression at the species (or morph) level [20, 21]. However, we currently know far less about whether individuals, when reacting to another species, are capable of adjusting their aggression according to differences in threat levels posed by different individuals of that species [22], or how familiarity or opportunities for learning may affect the adjustment of such aggression [5]. Accordingly, in a field-based experiment, we tested the influence of phenotypic similarity on aggression, as directed by breeding territory holders towards heterospecific ‘intruders’ in cichlids living in Nicaraguan Crater Lake Xiloá. Our study focused on two species of territory holders, *Amphilophus sagittae* and *Hypsophrys nicaraguensis*, which differed in their phenotypic similarity to the allopatric intruder species, *Amphilophus astorquii*, with which they were presented. The intruder species used in our study is allopatric with the two focal species, allowing us to control for any behavioural differences that might arise due to prior experience with the stimulus.

**Fig. 1:**
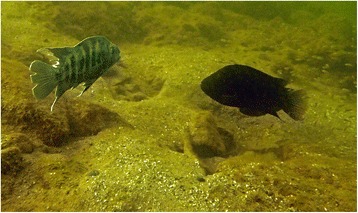
*Amphilophus sagittae* territory holders attacking a model of *A. astorquii* with non-breeding body markings. The male is closer to the camera, with the female only partially visible behind him

**Fig. 2:**
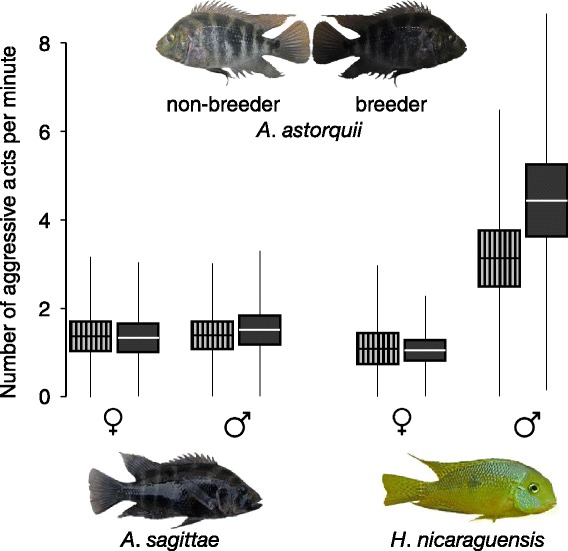
The total rate of aggression by *A. sagittae* and *H. nicaraguensis* territory holders (a specimen of each of the two species is pictured at the *bottom* of the graph) towards non-breeding (*boxes with vertical stripes*) versus breeding (*solid dark boxes*) intruder models of *A. astorquii* (pictured on the *top* of the graph). The results are given separately for the two sexes of the territory holders. Central *horizontal lines* within the boxes indicate means, *margins* of the boxes are for standard errors, and *whiskers* indicate standard deviations. Sample size for each box: *n* = 28

When ready to spawn, pairs of these cichlid fish species claim a sedentary breeding territory, which they aggressively defend for approximately a month after their fry have become free-swimming [23–26]. This aggression is directed towards both competitors (especially for territory space) and brood predators that can be conspecific, congeneric, as well as more distantly related species [25, 27]. In this respect, not all intruders pose the same level of threat. For instance, breeding individuals are likely to represent a lower threat than non-breeders, as the former have already claimed, and are busy defending, a territory and offspring of their own (and relying on previously accumulated energy reserves to do so), instead of actively seeking prey. In contrast, non-breeding individuals are much more likely to attempt to prey upon eggs and juveniles of both conspecifics and heterospecifics [22, 25, 26, 28]. Supporting this scenario, an earlier observational study of fish in Crater Lake Apoyo (Nicaragua) showed that non-breeding *A. astorquii* are subjected to more intense aggression than breeders, by both conspecific and congeneric (*Amphilophus zaliosus*) territory holders [22]. Importantly, breeding and non-breeding individuals (both males and females) of *A. astorquii*–as well as those of *A. sagittae* and *A. xiloaensis* in Crater Lake Xiloá–have strikingly different body markings: in contrast to the uniformly dark colour of the breeders, non-breeders have contrasting dark and light vertical bars along their flanks ([22]; Figs. 1 and 2). In addition, another recent study shows that for *A. sagittae* territory holders, colour patterning alone is a sufficient cue for directing more aggression towards model intruders with non-breeder colour markings than with breeder coloration, when these are look-alikes of the sympatric and congeneric species *A. xiloaensis* [19]. In contrast to the above-mentioned *Amphilophus* species, our other focal territorial species, *H. nicaraguensis*, does not exhibit any clear differences in body markings between breeding and non-breeding phases. It does, however, share the breeding habitat with multiple *Amphilophus* species (including *A. sagittae*), and is likely to be subject to similar ecological pressures. In this shared environment, *H. nicaraguensis* has to compete for territory space with *Amphilophus* species, and also defend its juveniles against them ([25], personal observations). Compared to the two *Amphilophus* species, *H. nicaraguensis* also has much more pronounced sexual size dimorphism, with males of *H. nicaraguensis* often reaching the size of small adult *A. sagittae* (≥15 cm total length), while female *H. nicaraguensis* are considerably smaller (typically below 10 cm) [25, 29]. Male and female *H. nicaraguensis* also have slightly different colour markings, with the latter possessing a prominent dark lateral stripe. By contrast, male and female *Amphilophus* do not differ in coloration, although males within a pair are slightly larger [25, 27, 29] and tend to have longer fin filaments than females.

Breeding and non-breeding individuals posing different threat levels to territory holders allowed us to test whether territory holders adjust their aggression depending on the presumed threat status of heterospecific intruders. We hypothesised that body colour patterns related to differences in breeding status in *A. astorquii* intruders would more likely influence territorial aggression in the congeneric and phenotypically similar *A. sagittae* compared to the dissimilar *H. nicaraguensis* (Fig. 2). Specifically, if recognition of intruder breeding status (based on coloration) is stronger when the intruder is phenotypically similar (see [2]), we would expect *A. sagittae* territory holders to make a clearer distinction between *A. astorquii* intruders differing in breeding status than *H. nicaraguensis* territory holders. The use of an allopatric intruder, *A. astorquii* (which is endemic to Lake Apoyo), controlled for the opportunity for stimulus learning. This is relevant because familiarity is known to often affect aggression in general [30–32], with opportunities for learning potentially also influencing heterospecific aggression [8, 9]. Similarly, if the sexes differ in their sensitivity towards the type of intruder (e.g. due to different abilities in heterospecific recognition, sensu Ord et al. [5]), we would also expect to see the pattern of responses to the different intruder types to be sex-specific (i.e. we would expect to find evidence of a sex × intruder type interaction).

## Methods

This field-based study was conducted using SCUBA in Lake Xiloá, Nicaragua (12°12.8′ N; 86°19.0′ W) between December 2013 and January 2014, during the breeding season of our two focal species, *Amphilophus sagittae* and *Hypsophrys nicaraguensis* ([23, 24], personal observations). In particular, we investigated the effect of phenotypic similarity on the level of territorial aggression towards non-breeders vs. breeders, while controlling for the opportunity for stimulus learning by using an allopatric intruder, *A. astorquii*. In addition, we explicitly controlled for any effects that might otherwise be caused by the behaviour of the stimulus, by using intruder models (or ‘dummies’). Such models have been successfully used to study behaviour in a range of fish species (reviewed by Rowland [33]), including *Amphilophus* [18, 19, 34] and other cichlids [35–37]. Instead of the stylised fish models that have been used in past studies [33–35], we opted for more realistic-looking models based on photographs of wild-caught fish following the methods of Lehtonen [18]. Specifically, we glued a waterproof, photographic colour print of the lateral side of a live or freshly euthanized specimen (sex unknown or not noted) onto each lateral side of an elliptical floating plate with a thickness of 6 mm. All our models were l6 cm long and attached to a sinker with a thin, transparent fishing line, so that they floated in a natural position approximately 15 cm above the lake bottom during the trials ([18, 19, 38]; Fig. 1). Models of this size were easy to handle under water and represented an overlap in the size ranges of adult male and female *A. sagittae* [19] as well as male *H. nicaraguensis*.

In our model presentation (see below), the breeder and non-breeder models (based on 24 different *A. astorquii* individuals) were paired. In 12 model pairs, the non-breeder model was, as explained above, based on a photo of a non-breeding individual, which was then also used to generate a ‘breeding’ counterpart by manipulating the image in Adobe Photoshop (Adobe Systems, Inc., San Jose, CA, USA) until it resembled a fish with the uniform (i.e. non-barred) markings of a breeding individual. Each ‘breeder’ counterpart had therefore exactly the same shape and posture as compared to its ‘non-breeder’ model pair, with colour markings being the only difference between the two models. For the rest (*n* = 12), in turn, the non-breeder counterpart model was made by manipulating the image of a breeding individual (in terms of shading and contrast) so that the horizontal bars became visible and the fish resembled a non-breeder (Figs. 1 and 2). Hence, in total we had 24 fixed model pairs, with each model pair made using a photograph from a different individual. These 24 models pairs were presented to 28 *A. sagittae* and 28 *H. nicaraguensis* territory-holding pairs defending small fry in a habitat characterised by pebbles lying on a finer substratum of sand and organic material. As a result, four model pairs were used twice for both focal species, while the rest of the models were used only once per species. This design was accounted for in the statistical analyses (see below).

Each trial started by placing an *A. astorquii* model approximately 40 cm from the centre of the focal territory, which is at, or slightly below, an average distance territory holders swim when deterring territorial intruders [22, 27, 39]. We counted the total number of aggressive responses (either slow movement toward the model with flared gills and fins in a threat display or a rapid advance sometimes followed by a bite [18, 19]) by both male and female territory owners towards the model for 5 min (giving the total aggression rate sensu [19, 27]). We then removed the model from sight for a predetermined rest period of 5 min, after which we repeated the above with the alternative model (i.e. a breeder model if the initial model was a non-breeder and vice versa). The model type (breeder versus non-breeder) presented first was randomised. After the trial, the territory was marked with a numbered piece of ceramic tile to avoid assessing the same territory more than once.

### Statistical analyses

To assess the effects of the species (*A. sagittae* versus *H. nicaraguensis*) and sex (male versus female) of the focal territory holder, as well as the type of model intruder (breeder versus non-breeder), we applied a generalized mixed model using the ‘glmmpql’ function of the packages ‘nlme’ and ‘MASS’ with a negative binomial error distribution appropriate for over-dispersed count data [40]. To account for the non-independence of the actions of a territory-holding male and female, as well as any effects related to the use of stimulus model pairs in more than one replicate, ‘breeding pair/territory ID’ and ‘model pair ID’ were added as random effects (as per [41]). We then proceeded with stepwise refits of the model, each time without its least significant, highest order interaction term, using *p* = 0.05 as the cut-off point. We used R 3.2.2 software (R Development Core Team) for all analyses.

## Results

When we applied a generalized mixed model to assess the effects of the species and sex of the focal territory holders and the ‘breeder/non-breeder’ status of the model intruder on the rate of aggression, we found a significant interaction between focal species and sex (t_219_ = 6.38, *p* < 0.001): male *H. nicaraguensis* exhibited a higher rate of aggression than females, whereas there was no pronounced sex difference in *A. sagittae* (Fig. 2). The effect of intruder status (i.e. breeder versus non-breeder colour markings) was not significant (t_219_ = 1.05, *p* = 0.29) (Fig. 2). We also considered the possibility that our results might have been affected by the artificial manipulation of our models (i.e. image manipulation in Photoshop). We assessed this by reanalysing the data comparing only the aggressive responses towards *A. astorquii* models that exhibited natural (i.e. non-manipulated) breeding versus non-breeding colour patterns. The results, however, remained qualitatively the same: there was an interaction between sex and species (t_107_ = 4.20, *p* < 0.001), whereas the status of the model (i.e. breeder versus non-breeder) did not have a significant effect (t_107_ = 0.818, *p* = 0.41).

## Discussion

We found that neither *H. nicaraguensis* nor *A. sagittae* territory holders reacted differently to breeder vs. non-breeder model intruders of *A. astorquii*. In other words, contrary to our expectation, neither of the two focal species adjusted their aggression to the allopatric heterospecific signal. Earlier studies using both manipulated [19] and natural [22] stimuli have nevertheless strongly indicated that *Amphilophus* cichlids do react differently to sympatric breeders and non-breeders, with coloration (of model intruders) being a sufficient cue for aggression level adjustments in both of our focal species [18, 19, 38]. Below we discuss why we did not find adjustment of aggression towards the different models of *A. astorquii* intruders in the current study.

First, we consider the possibility that one or both focal species had the capacity to correctly distinguish between breeder and non-breeder (model) intruders but chose not to modify their aggression because the signal was not clear or sufficiently relevant to induce a response. In the case of *H. nicaraguensis*, it remains possible, for example, that differences in the motivations of intruders of the more distantly related *Amphilophus*, as displayed by the breeding and non-breeding colour patterns, are not relevant enough for territory holders to significantly adjust their aggressive behaviour. However, this possibility is less likely to explain why the closely related and phenotypically similar *A. sagittae* territory holders also did not respond differently to the two breeder types, even though they do direct more aggression towards models of breeders compared to non-breeders of the sympatric *A. xiloaensis* [19]. It is nevertheless feasible that to avoid any costs of misplaced aggression more generally, both species may have evolved, or territory holders may have learned, not to modify their territorial aggression when the stimulus cues do not match well enough with the specific signals that are displayed by conspecifics or phenotypically similar species with which they are sympatric. Next, we consider proximate mechanisms that could have resulted in the lack of response to the allopatric breeding status signal. In other words, we consider the possibility that the territory holders might simply have not succeeded in making the distinction between breeders and non-breeders when these were allopatric.

At the proximate level, it is feasible that mere differences in markings and colour brightness between allopatric breeders and non-breeders, without any supporting behavioural differences, may have given too subtle a cue for the territory holders to adjust their aggression. In other words, because the ability to distinguish between breeders and non-breeders [19, 22] has, by default, evolved in interaction with species sharing the same environment (i.e. sympatric species), the territory holders may not be able to recognise the equivalent cues when signalled by allopatric species. This possibility supports the hypothesis and empirical observations that interactions with non-native competitors or predators can result in inappropriate behavioural responses [27, 42–44]. For instance, the results reported in the current study are consistent with an earlier study investigating the response of *Amphilophus zaliosus* parents towards an introduced predator, the bigmouth sleeper (*Gobiomorus dormitor*) in Lake Apoyo [27]. That study showed that fry-guarding parents allowed the non-native predator to venture much more closely to their fry before reacting to them compared to the distance that native fish predators were allowed to approach. Hence, signals used to recognise competitors or predators may result in inappropriate behavioural responses when individuals are exposed to novel or unfamiliar signals, which can have negative fitness consequences for the receiver and/or benefit the novel (invasive) species [42, 45]. In this respect, if our results are due to a failure of the focal Lake Xiloá residents in recognising the breeding status signal of allopatric *A. astorquii* intruders, we do not currently know whether the observed response would have been an overreaction to breeders or an underreaction towards non-breeders. In the case of an actual invader, a likely consequence of the former would be increased energy expenditure, whereas the latter could result in increased rates of predation on eggs and juveniles (see [27, 44]).

Finally, we consider the possibility that our models simply did not accurately represent differences between *A. astorquii* breeders vs. non-breeders. In this respect, we prepared our models by adjusting shading and contrast of one model in each pair to mimic the patterns of the opposite breeding status. However, we do not believe that this artificial manipulation of colour patterns per se explains the results. This is because even when we analysed the reactions towards *A. astorquii* models with natural breeding and non-breeding coloration, we still found no difference in response towards the two colour types. Furthermore, our models were thinner than actual fish, and we therefore cannot rule out the possibility that territory holders may have perceived the models as individuals in poor body condition. If this was the case, territory holders might have regarded the models as a lower threat compared to living intruders in good condition. However, it is important to point out that earlier studies have demonstrated significant aggression adjustments to colour differences in similar intruder models (i.e. with a thickness of 6 mm) of sympatric species [18, 19, 38].

Due to the argument of Ord et al. [5] that sex differences in the opportunity or ability to learn relevant cues may induce differences in heterospecific recognition between males and females, we also assessed differences between the sexes in their reactions towards breeder versus non-breeder models, when deliberately controlling for learning opportunities by using an allopatric stimulus. Our results do not provide evidence for sex differences in recognition (or relevance) of the allopatric signal. We did nevertheless find an overall sex difference in aggressiveness in *H. nicaraguensis* but not in *A. sagittae*. This result is likely to reflect a general difference in sex roles between these two species. In particular, it seems that in *H. nicaraguensis*, more so than in *A. sagittae*, males and females have evolved divergent roles in territory defence. Specifically, we found that *H. nicaraguensis* males were far more aggressive compared to females, whereas aggressive responses were much more evenly distributed between the sexes in *A. sagittae*. We note that the size of our intruder models (total length: 16 cm) relative to self may have been perceived more similarly between the sexes in *A. sagittae* as compared to *H. nicaraguensis*, given that males are only slightly larger than females in the former (typical male standard length: 13–18 cm, typical female standard length: 10–15 cm) but much larger than females in the latter (typical male standard length: 8–12 cm, typical female standard length 4–8 cm) [25, 29].

## Conclusion

We found that although the two focal species share the same breeding habitat and are likely to be subject to similar ecological pressures in the shared environment, they nevertheless exhibited different sex roles in breeding territory defence. This means that different species have evolved divergent approaches for successful parental care. However, we did not find evidence for differentiation between sexes in the pattern of aggression adjustment in either species. Interestingly, we found that when the stimulus was allopatric, aggressive defence of the breeding territory was not adjusted towards stimuli with contrasting breeding status coloration, independent of the phenotypic similarity between the territory holders and intruders. This is in contrast to earlier studies that used similar methodology but with sympatric (rather than allopatric) intruder stimuli. When considered together with these earlier findings, the current results underscore the importance of considering familiarity and coevolution in heterospecific competitor recognition.

### Ethics

The study was approved by MARENA (Ministerio del Ambiente y los Recursos Naturales, Nicaragua: permit no. 013-102013) and is compliant with all relevant laws for the ethical treatment of animals in scientific research.

### Availability of supporting data

Our data have been uploaded to Dryad: http://dx.doi.org/10.5061/dryad.79p2b.
